# A High-Yield Two-Hour Protocol for Extraction of Human Hair Shaft Proteins

**DOI:** 10.1371/journal.pone.0164993

**Published:** 2016-10-14

**Authors:** Sing Ying Wong, Ching Chin Lee, Ali Ashrafzadeh, Sarni Mat Junit, Nazirahanie Abrahim, Onn Haji Hashim

**Affiliations:** 1 Department of Molecular Medicine, Faculty of Medicine, University of Malaya, 50603 Kuala Lumpur, Malaysia; 2 Medical Biotechnology Laboratory, Faculty of Medicine, University of Malaya, 50603 Kuala Lumpur, Malaysia; 3 University of Malaya Centre for Proteomics Research, Faculty of Medicine, University of Malaya, 50603 Kuala Lumpur, Malaysia; Centre National de la Recherche Scientifique, FRANCE

## Abstract

Proteome analysis of the human hair remains challenging due to the poor solubility of hair proteins and the difficulty in their extraction. In the present study, we have developed a rapid extraction protocol for hair shaft protein using alkaline-based buffer. The new protocol accelerated the procedure by reducing the extraction time from at least a day to less than two hours and showed a protein recovery of 47.3 ± 3.72%. Further analyses of the extracted protein sample through sodium dodecyl sulfate polyacrylamide gel electrophoresis and Quadrupole-time-of-flight mass spectrometry analysis unveiled a total of 60 proteins, including 25 that were not previously reported. Identification of these proteins is anticipated to be crucial in helping to understand the molecular basis of hair for potential applications in the future.

## Introduction

The human hair shaft consists of three different layers: medulla being the innermost layer, cortex being the middle part, and the outermost layer which is a scaly layer known as the hair cuticle [1]. The major hair proteins, keratins and keratin associated proteins (KAPs), have been shown to be expressed specifically in the respective layers of the hair shaft [2]. These proteins are very stable and resistant to enzymatic degradation [3] and therefore has a strong potential to be used as markers for various purposes. The study of human KAPs has advanced significantly in recent years. However, it remains tough and challenging due to its high keratin content and vast transglutaminase-mediated isopeptide cross-linking that prevents solubilization of approximately 15% of the constituent protein even in the presence of strong denaturants [4].

Different methods for extraction of proteins from hair samples have been previously reported. One of the most commonly used protocols is known as Shindai method [5] which was developed based on the procedure used by Fujii and coworkers to extract protein from hair, nail, and wool. The protocol involves incubation of the samples at 50°C for 24 h in a buffer consists of 20 mM Tris-HCl (pH 8.5), 2.6 M thiourea, 5 M urea, and 5% (v/v) 2-mercaptoethanol (2-ME), known as Shindai solution. The Shindai method was subsequently modified for effective purification of KAPs from the human hair by addition of various low molecular weight alcohols to the Shindai solution and without using 2-ME or sodium dodecyl sulfate (SDS) [6]. Recoveries of the KAPs and keratin fractions from the hair samples using the Shindai method were approximately 10 and 50%, respectively.

In another study by Barthelemy and co-workers [7], a similar extraction solution with the addition of 0.1% Triton X-100 and incubation at 37°C for 18 h, was utilized. However, the recovery of proteins was not stated in the report. Instead, nanoscale liquid chromatography coupled to tandem mass spectrometry (NanoLC-MS/MS) was used to analyze the extracted proteins, leading to identification of 56 proteins, including 34 keratins and KAPs.

The protocols used by Barthelemy et al. [7] and the earlier mentioned Shindai method [5] had both used urea as a protein denaturant. In a study performed by Kollipara and Zahedi [8], one fifth of N-termini of proteins and approximately 2% of their Lys residues were shown to be carbamylated during overnight incubation in presence of 2.0 M urea at 37°C. Hence, the group suggested that the usage of urea had to be completely avoided in all sample processing steps.

Nevertheless, an extraction method of hair shaft proteins without the use of urea-containing buffer has been earlier reported. Lee and coworkers [9] incubated hair samples in a solution consisting of 2% SDS, 50 mM sodium phosphate (pH 7.8), and 20 mM dithiothreitol (DTT) and incubated overnight at 65°C. The results showed that the first two SDS-DTT treatments were able to remove approximately 80% of the total protein extracted. Besides, the study also reported that in six experiments, the final insoluble material comprised 13.3 ± 3.9% of the total protein. However, the exact percent recovery of the total protein extracted was not mentioned in this study. Whilst the recovery of proteins was not mentioned in their report, the study has successfully identified over hundreds of proteins using Multidimensional Protein Identification Technology (MudPIT).

The present study was aimed at developing a rapid method for extraction of high yield proteins from the human hair shaft and compares it to the previously reported methods in terms of their percent recovery and efficiency. Development of an improved protocol in the extraction of hair shaft proteins may open new doors to study their roles in molecular pathways and their potential applications as markers.

## Materials and Methods

### Preparation of hair samples

Samples of hair shaft were collected from 10 unrelated volunteer subjects with an equal number of males and females whose ages ranging from 21 to 40 years. Subjects were excluded if they had hair or scalp problems including skin inflammation, skin cancer and bacterial or fungal infection. Individuals whose hair was chemically treated were also excluded. Written informed consent was obtained from all participants prior to sample collection. This study and its consent procedure were approved by the Ethical Committee of the University of Malaya Medical Centre (UMMC) (Institutional Review Board) in accordance with the ICH-GCP guideline and the Declaration of Helsinki (Ref. no.: UM.TNC2/RC/2H&E/UMREC-64). The hair samples were sterilized with 90% ethanol and cut into approximately 1 cm in length with a pair of sterile laboratory scissor before incubated in lysis buffer.

### Hair protein extraction

#### Extraction conditions

Five mg of the 1 cm-long hair sample was treated in 300 μl of lysis buffer which contained NaOH (0.1 M and 0.2 M), and sodium dodecyl sulfate (SDS; 1% and 2%), beta-mercaptoethanol (β-ME, 2%) and ethylenediaminetetraacetic acid (EDTA, 0.01 M). All mixtures were incubated at 90°C for 10 minutes (Table 1). The mixture was transferred into QIAquick Spin Columns (Qiagen) and centrifuged at 16000 rpm for 5 minutes to separate undissolved hair fractions and supernatant. The protein sample was further purified and precipitated by mixing 1 volume of the supernatant with 4 volumes of pre-cold acetone. The precipitated protein was reconstituted in a sample buffer contained 7 M urea, 2 M thiourea, 4% 3-[3-Cholamidopropyl)dimethylammonio]-1-propanesulfonate (CHAPS), 2% ampholytes and 5% glycerol. All reconstituted protein samples were later stored at 4°C. The amount of protein recovered from each experiment was measured by Bradford colorimetric method [10]. The percentage of protein recovery was calculated based on the total extractible hair proteins of each individual, which was the whole fibre weight of the hair sample used. Lysis buffer that produced highest protein recovery was chosen for subsequent experiments.

**Table 1:** Effects of SDS and NaOH on recovery of human hair shaft proteins.

Reagents*	Temperature (°C)	Time (min)	Recovery (%)
0.1 M NaOH	90	10	0
0.2 M NaOH	90	10	0
1% SDS	90	10	0
2% SDS	90	10	0
0.1 M NaOH and 1% SDS	90	10	7
0.1 M NaOH and 2% SDS	90	10	8
0.2 M NaOH and 1% SDS	90	10	19
0.2 M NaOH and 2% SDS	90	10	19

*all reagents also contained 2% β-ME, and 0.01 M EDTA

Samples of 5 mg were immersed in the formulated alkaline-based lysis buffer and incubated for 5, 10, 20, 30 and 40 minutes at 90°C. A tube of 5 mg of hair similarly immersed in lysis buffer was also set up as control of the experiment and left at room temperature for 60 minutes. The mixtures were processed and the protein concentration of each sample was measured as earlier described. A graph was plotted based on the average weight recovery (%) of hair protein from all ten individuals to determine the optimal incubation time. Approximately 100 μg of protein sample obtained from each incubation period was separated by electrophoresis in over a 12% sodium dodecyl sulfate polyacrylamide gel (SDS-PAGE). The SDS-PAGE was performed on the Bio-Rad mini-gel system at 150 V for 2 hours. The gel was silver stained as previously described [11] and finally scanned with the ImageScanner III and analyzed using GelAnalyzer 2010 software.

#### Additional protein extraction

To maximize protein recovery from the human hair shaft, extraction was carried out in two stages. The first stage of extraction was carried out according to the conditions tested in the earlier experiments, where hair sample from an individual was immersed in 300 μl of formulated alkaline-based lysis buffer and incubated based on the optimal time obtained at 90°C. During the second stage of extraction, undissolved hair fractions from the previous extraction were transferred using a pair of sterile forceps into a fresh tube containing 300 μl of lysis buffer with different concentrations of NaOH (0.1, 0.15 and 0.2 M).

After obtaining the optimal concentration of NaOH, a different approach which involved mechanical forces by magnetic stirring was introduced to increase the recovery of protein from the second stage of extraction. Leftover hair fractions from the first stage of extraction were transferred into 300 μl alkaline lysis buffer containing suitable NaOH concentration. It was allowed to pulverize by magnetic stirrer at room temperature. The solutions were filtered, precipitated and estimated for protein concentration using the method of Bradford, and recovery was calculated based on the whole fibre weight of hair. The efficiency of the method with and without magnetic stirring was compared.

After attaining the most suitable protein extraction method for both stages of extraction, the protocol was carried out in ten individuals to calculate the average percent recovery. SDS-PAGE separation was also performed to compare the concentration of proteins obtained from the two stages of extraction. Proteins obtained from both extracts of an individual’s hair were identified by mass spectrometry and database searches. The conditions used in running SDS-PAGE were as mentioned previously.

### Comparison of protein extraction methods

Aside from using the alkaline-lysis method developed in the present study, extraction of hair proteins was also performed using the previously described methods of Fujii et al. (i.e., Shindai method) [5] and Lee et al. [9]. In the Shindai method, hair samples were immersed in 300 μl of lysis buffer that composed of 20 mM Tris-Hydrochloric acid (Tris-HCl) (pH 8.5), 2.6 M thiourea, 5 M urea and 5% (w/v) 2-mercaptoethanol at 50°C for 24 h and 48 h [5], whilst in the method developed by Lee and co-workers [9], hair samples were rinsed briefly in 300 μl of lysis buffer containing 2% SDS, 50 mM sodium phosphate (pH 7.8) and drained. The hair was later immersed in the same lysis buffer with addition of dithiothreitol (DTT, 20 mM) and incubated overnight at 65°C. It was pulverized by magnetic stirrer for an hour at room temperature, and the soluble and insoluble materials were separated by centrifugation. The insoluble material was again immersed in the same buffer and incubated overnight. This was repeated for another four times to extract as much insoluble proteins as possible [9], prior to being filtered and precipitated. Protein solutions obtained from the same individual using the three different methods of extractions were finally measured and compared using the colorimetric method of Bradford [10].

### MS Analysis

Bands from SDS-PAGE gels, which were instead stained with Coomassie Brilliant Blue R250, were excised using a sterile scalpel. The gel was excised as close to the band as possible, with no excess around it. The entire lane was cut into 8–12 slices, regardless of the staining profile. The bands were then cut into 1 x 1 mm pieces and kept into a 1.5 ml Lobind Eppendorf tube. After excising the gel bands, reduction and alkylation of proteins were performed to prevent the disulphide bonds formation using DTT and iodoacetamide, respectively. In gel tryptic digestion was performed using 6 ng/μl trypsin in 50 mM ammonium bicarbonate (Sigma, Steinheim, Germany) overnight at 37°C as a part of the sample preparation for the mass spectrometric identification of proteins. Extraction of peptides from the gel slices was initially performed using 50 μl of 50% acetonitrile, and subsequently 50 μl of 100% acetonitrile, that were lyophilised in a vacuum centrifuge. Finally, Ziptip-C18 column (Millipore) clean-up was done prior to protein identification.

Peptides were then reconstituted in 10 μL of 0.1% formic acid. Peptide separation was performed by 1260 Infinity Nanoflow LC system (Agilent, Santa Clara, CA, USA) directly connected to Accurate-Mass Q-TOF 6550 with a nano electrospray ionization source. Briefly, the peptides were separated over an HPLC Large-Capacity Chip Column (Zorbax 300SB-C18, 160 nL enrichment column, and 75 μm × 150 mm analytical column, and 5 μm particles, Agilent, Santa Clara, CA, USA) with a 5–70% linear gradient of solvent B (0.1% formic acid in 100% acetonitrile) for 15 min with a flow rate of 0.4 μL/min. Mass spectra were acquired using Mass Hunter acquisition software (Agilent, Santa Clara, CA, USA). Each mass spectra acquisition cycle (5.225 seconds with an acquisition rate of 20 spectra per second from 200 to 3000 m/z) was followed by collision-induced dissociation of the twenty most intensive ions. MS/MS data were acquired in the range of 50–3200 m/z.

### Database search

Spectrum Mill software (Agilent, Santa Clara, CA, USA) was set to search MS/MS acquired data against Swiss-Prot databases (updated 3^rd^ December 2015) human (*Homo sapiens*) (168 628 entry sequences). Mass-tolerance of precursor and product ions was set to ± 20 and ± 50 ppm respectively. As iodoacetamide was used for alkylation purposes during sample preparation, carbamidomethylation was specified as a fixed modification and oxidized methionine as a variable modification. The precursor mass shift was set between -18 Da to 177 Da, to take into consideration of variable modifications such as presence of sodium and potassium adducts. Proteins or peptides that were identified were validated using Spectrum Mill, based on the software default settings. Inclusion criteria included protein score of more than 20, peptide score > 10 and Scored Peak Intensity (% SPI) > 70%. Proteins that shared at least one peptide were grouped together. Identified proteins were then filtered to achieve a false discovery rate (FDR) of < 1% for the peptide-spectrum matches.

Scaffold (version Scaffold_4.4.6, Proteome Software Inc., Portland, OR) was used to validate Spectrum Mill based peptide and protein identifications using x-tandem algorithm (The GPM, thegpm.org; version CYCLONE (2010.12.01.1)). Peptide identifications were accepted if FDR of less than 0.1% could be established by the Peptide Prophet algorithm [12]. Protein identifications were accepted if more than 95.0% probability could be established and the protein contained at least 2 identified peptides. Protein probabilities were assigned by the Protein Prophet algorithm [13]. Proteins sharing significant peptide evidence were grouped into clusters and the exclusively unique peptides of each protein were identified.

## Results

### Formulation of alkaline lysis buffer

Table 1 shows the effects of SDS and NaOH on efficiency of extraction of the human hair shaft proteins. In this experiment, the temperature and time of incubation were kept constant at 90°C and 10 minutes respectively, while the reagents and concentrations of SDS and NaOH were altered in presence of 2% of β-ME and 0.01 M EDTA. No protein was recovered in the lysis buffer containing only SDS or NaOH. The extraction yield was increased from 8% to 19% when 0.2 M NaOH instead of 0.1 M NaOH was used in the experiment. Meanwhile, an increase of the concentration of SDS from 1% to 2% in 0.01 M of NaOH resulted in minimal increased of percent recovery by 1%. Highest percent of recovery was attained with the use of buffer containing 0.2 M NaOH, 1% of SDS, 2% of β-ME and 0.01 M EDTA.

### Determination of optimal incubation time

**Fig 1:**
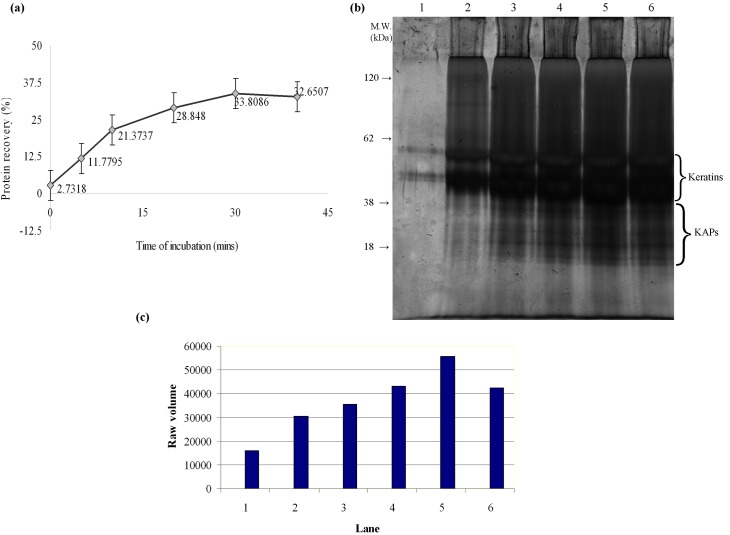
Temporal progress of incubation time for extracting proteins from the human hair shaft. (a) Graph shows the percent recovery of protein obtained when hair was incubated for 5, 10, 20, 30 and 40 minutes at 90°C. Minute 0 acts as the control of the experiment, in which the incubation mixture was left at room temperature for 60 minutes. Each value represents the average percent recovery of ten individuals. (b) Representative SDS-PAGE gel showing proteins extracted using the present method when hair of an individual was incubated from 5–40 minutes in alkaline lysis buffer at 90°C, alongside with the control. Lane 1 represents control. Lanes 2 to 6 show the bands produced from 5, 10, 20, 30 and 40 minutes, respectively. (c) Raw volume of the keratin bands (Lanes 1–6) obtained from the representative figure of gel from SDS-PAGE.

Panel (a) of Fig 1 demonstrates the temporal progress of incubation time for extraction of proteins from the human hair shaft of 10 individuals using buffer containing 0.2 M NaOH, 1% of SDS, 2% of β-ME and 0.01 M EDTA. Hair (5 mg) was incubated at different periods of incubation (5, 10, 20, 30, and 40 minutes) to determine an optimal time to obtain the greatest yield of protein. The recovery for the control was 2.7 ± 1.02% when the incubation mixture was left at room temperature for an hour. Statistical analysis was performed using Kruskal-Wallis test and significant differences (p < 0.05) were observed between control to 20 minutes, control to 40 minutes, control to 30 minutes, 5 to 20 minutes and 5 to 30 minutes. The degree of extraction has reached a plateau by 30 minutes, with 33.8 ± 0.61% of protein recovery at 30 minutes and 32.6 ± 0.65% at 40 minutes. No significant difference was found in the protein recovery from 20 to 30 minutes and from 30 to 40 minutes of incubation. The extracted hair shaft proteins were then subjected to SDS-PAGE (Fig 1, panel (b)) and the bands produced were analyzed using GelAnalyzer 2010 software. Four different sample buffers, including (a) Laemmli buffer, (b) Tris-HCl pH 7.8, (c) Tris-saline pH 8.0 (150 mM sodium chloride, 1% Nonidet P-40/Triton X-100, 50 mM Tris) and (d) Alkaline lysis buffer (7 M urea, 2 M thiourea, 4% 3-[(3-Cholamidopropyl) dimethylammonio]-1-propanesulfonate (CHAPS), 2% ampholytes and 5% glycerol) were initially tested in an attempt to completely dissolve the acetone-precipitated pellets, prior to the SDS-PAGE. Our results showed that the pellets not only appeared to completely dissolve only in the alkaline lysis buffer, but also did not form precipitate when stored at 4°C. Fig 1, panel (c) shows the increase of raw volumes of the bands with longer incubation periods, with 30 minutes being the highest, which then declined at 40 minutes of incubation time.

### Extraction of additional proteins

In the following experiment, attempts were made to increase recovery by performing additional extraction using the leftover fractions of hair. In the first stage of extraction, proteins were similarly extracted using 0.2 M NaOH and incubated at 90°C for 30 minutes, resulting in an average protein recovery of 45.22 ± 2.3% (Table 2). In the second extraction, protein recovery declined when the concentration of NaOH was increased from 0.1 M to 0.2 M while the temperature of incubation was kept constant at 90°C. When the leftover hair fractions were pulverized in alkaline lysis buffer containing 0.1 M NaOH using a magnetic stirrer and at room temperature, highest protein recovery at 19 ± 0.17% was obtained (Table 2). Hence, the magnetic stirrer was used in all subsequent experiments as it appeared to enhance efficiency of extraction of proteins during the second stage of extraction. When taken together, the average total percent recovery of proteins from both extracts of 5 mg of hair shaft of 10 individuals was 47.3 ± 3.72%. This includes (i) extraction of proteins in the first stage, which had the ability to recover up to 33.8 ± 2.37% of hair weight, and (ii) subsequent extraction of the leftover fractions of human hair shaft, which resulted in an additional of 13.4 ± 1.92%.

**Table 2:** Comparison of protein recovered from the second stage of extraction by magnetic stirring and incubation method.

Methods	Temperature	Concentration of NaOH used in alkaline lysis buffer (M)	Protein recovery* (%)
Incubation (without magnetic stirring)	90°C	0.1	12.88 ± 0.79
		0.15	6.16 ± 0.87
		0.2	2.44 ± 0.64
Magnetic stirring	Room temperature	0.1	19.00 ± 0.17

*Each value of protein recovery (%) represents the average of three repetitive experiments using hair samples from the same individual.

### Comparison with Shindai method and the method of Lee et al.

**Fig 2:**
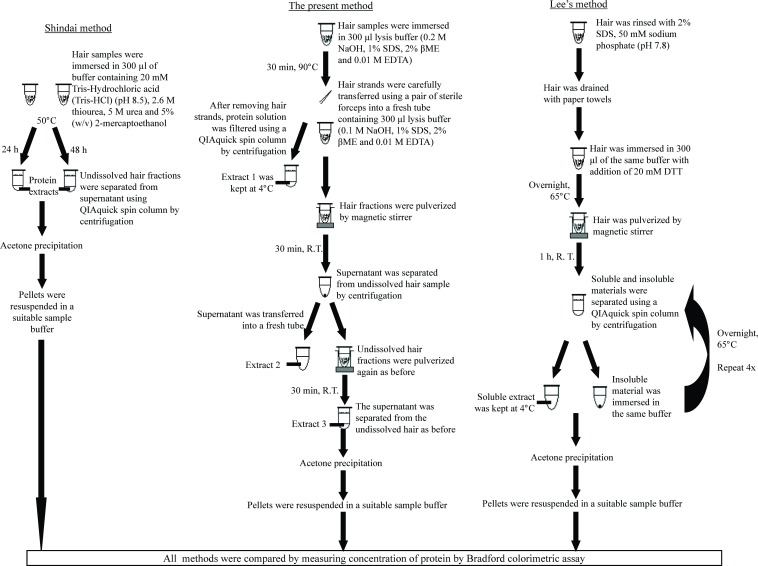
Outline of protein extraction procedures. The Shindai method [5], the alkaline lysis method developed in the present study and the method of Lee et al. [9] are illustrated.

The present study also compared the efficiency of protein extraction of the present method with the earlier developed protocols of Fujii et al. (i.e., Shindai method) [5] and that of Lee and co-workers [9]. The protocols involved in each of the protein extraction methods are illustrated in Fig 2. Table 3 shows the results of the experiments. Marked improvement was observed when our data was compared to the protein extraction protocols using Shindai method [5] and that of Lee et al. [9] for the same 5 mg weight of hair shaft. The rates of protein extraction were also calculated and compared between the three different extractions. For the alkaline lysis method, the rate of protein extraction was 43.17%/h, while the method of Lee [9] produced an extraction rate of 0.38%/h. On the other hand, the extraction rates of methods 1 and 2 of Shindai [5] were 0.974%/h and 0.336%/h, respectively.

**Table 3:** Recovery of hair shaft proteins using different methods of extraction.

Methods of extraction*	Time (hours)	Recovery (%)	Total recovery (%)
**Alkaline lysis method**	0.5	46.38	64.76
	1	17.84	
	1.5	0.54	
**Method of Lee et al. [9]**	24	17.15	45.99
	48	19.19	
	72	5.68	
	96	2.72	
	120	1.23	
**Shindai method 1 [5]**	24	23.38	23.38
**Shindai method 2 [5]**	48	16.13	16.13

*These experiments were performed using hair samples from the same individual.

### Identification of hair shaft proteins by mass spectrometry

**Fig 3:**
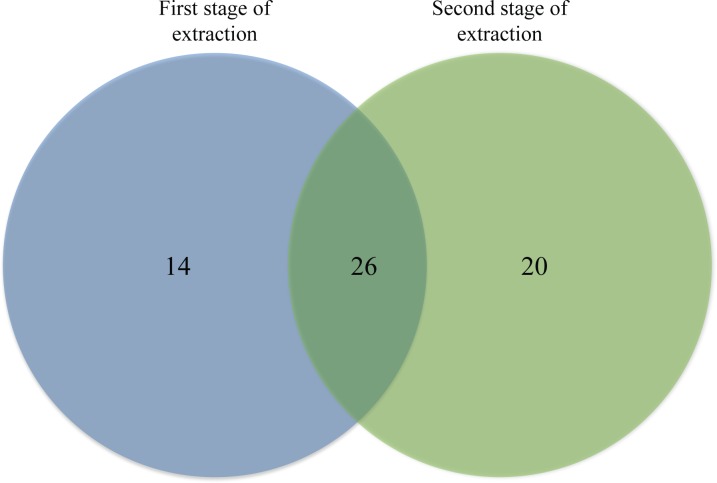
Venn diagram comparing the proteins identified from both stages of protein extraction.

**Fig 4:**
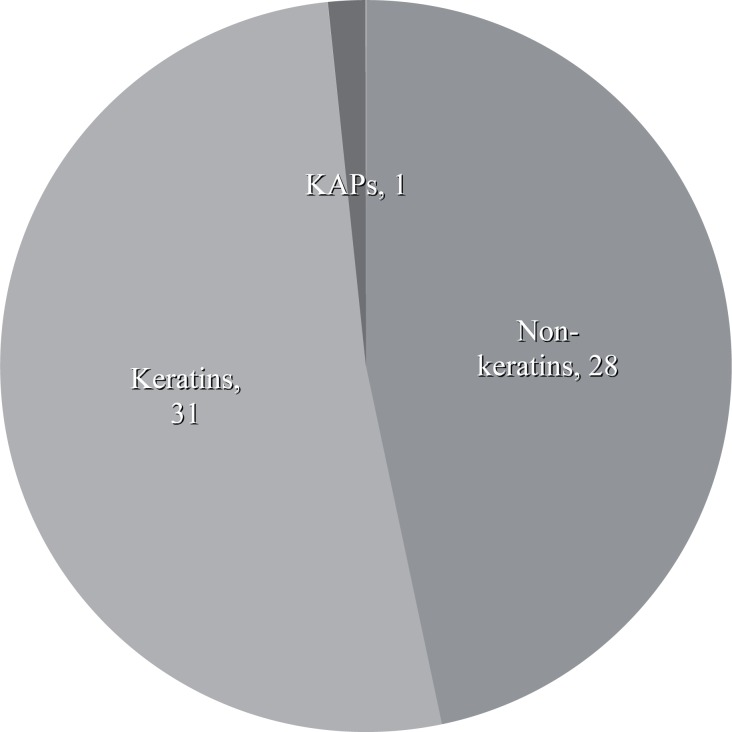
Pie chart showing the distribution of keratins, non-keratins and keratin-associated proteins (KAPs).

When the proteins extracted using the method developed in the present study was subjected to LC MS/MS Q-ToF analysis and database query, a total of 60 proteins were identified. Among the 60 proteins, 14 were found only from the first stage of extraction, while 20 were exclusively detected from the second stage of extraction (Table 4 and Fig 3). Both stages of protein extraction have also detected 26 common proteins (Fig 3). All the proteins identified were also categorized into non-keratin proteins, keratin proteins and keratin-associated proteins (Fig 4).

**Table 4:** Identification of hair shaft proteins.

Accession number	Proteins identified	No. exclusive unique peptides	
		First stage	Second stage
**(a) Proteins identified only from the first stage of protein extraction.**			
Q6A165	*HHa 7 Protein (Fragment)	1	-
A0JNT2	Keratin 83 protein	1	-
A6PVX1	Selenium-binding protein 1	1	-
Q9BYR8	Keratin-associated protein 3–1	2	-
P16104	Histone H2A	2	-
B4DPP6	*cDNA FLJ78413, highly similar to Homo sapiens albumin, mRNA	2	
Q5JW98	Isoform 3 of Protein FAM26D	3	-
A0A024RBK8	*RAS protein activator like 1 (GAP1 like), isoform CRA_b	2	-
P07355	Annexin	3	-
P15090	*Epididymis secretory protein Li 104	2	-
P31947	Isoform 2 of 14-3-3 protein sigma	3	-
B4DLJ7	*cDNA FLJ59334, highly similar to Transmembrane glycoprotein NMB	2	-
P06576	ATP synthase subunit beta, mitochondrial	2	-
V9HW26	ATP synthase subunit alpha	2	-
**(b) Proteins identified only from the second stage of protein extraction.**			
Q9NSB4	Keratin, type II cuticular Hb2	-	1
A8K2I0	cDNA FLJ78504, highly similar to Homo sapiens keratin 6A (KRT6A), mRNA	-	2
B4DN72	cDNA FLJ55910, highly similar to Keratin, type II cuticular Hb6	-	1
B7Z7N3	cDNA FLJ51392, highly similar to Keratin, type II cuticular Hb5	-	1
Q5XKE5	*Keratin, type II cytoskeletal 79	-	1
O76014	Keratin, type I cuticular Ha7	-	1
P02533	*Keratin, type I cytoskeletal 14	-	2
P08779	*Keratin, type I cytoskeletal 16	-	1
Q99456	*Keratin, type I cytoskeletal 12	-	1
B2R4P9	Histone H3	-	3
Q5VU13	V-set and immunoglobulin domain-containing protein 8	-	3
P15924	Desmoplakin	-	4
Q9ULI3	*Isoform 2 of Protein HEG homolog 1	-	2
P27482	Calmodulin-like protein 3	-	3
P35030	*PRSS3 protein	-	2
Q5IBP5	*AKAP9-BRAF fusion protein	-	2
Q8IVL0	*Isoform 2 of Neuron navigator 3	-	2
O95147	Dual specificity protein phosphatase 14	-	2
P36873	Isoform Gamma-2 of Serine/ threonine-protein phosphatase PP1-gamma catalytic subunit	-	2
C9JF17	*Apolipoprotein D	-	2
**(c) Proteins identified from both stages of protein extraction.**			
Q14525	Keratin, type I cuticular Ha3-II	10	10
O76011	*Keratin, type I cuticular Ha4	11	18
Q15323	*Keratin, type I cuticular Ha1	7	10
O76009	Keratin, type I cuticular Ha3-I	9	7
P13645	*Keratin, type I cytoskeletal 10	10	19
A0A024R1T2	Keratin, hair, acidic, 5, Isoform CRA_a	6	10
O76013	Isoform 2 of Keratin, type I cuticular Ha6	2	2
O76015	Keratin, type I cuticular Ha8	2	1
Q14532	Keratin, type I cuticular Ha2	1	1
A8K872	*cDNA FLJ77849, highly similar to Homo sapiens keratin, hair, basic, 6 (monilethrix) (KRTHB6), mRNA	4	1
Q14533	*Keratin, type II cuticular Hb1	3	2
P78385	Keratin, type II cuticular Hb3	1	7
P78386	Keratin, type II cuticular Hb5	33	13
H6VRG1	Keratin 1	17	23
O95678	*Keratin, type II cytoskeletal 75	2	2
P35527	*Keratin, type I cytoskeletal 9	9	14
B3KY79	*cDNA FLJ46620 fis, clone TLUNG2000654, highly similar to keratin, type II cytoskeletal 7	0	1
P35908	Keratin, type II cytoskeletal 2 epidermal	5	9
Q6A163	Keratin, type I cytoskeletal 39	4	7
V9HWG1	*Epididymis secretory sperm binding protein Li 134P	5	3
Q0VAS5	Histone H4	3	4
Q86SJ6	Isoform 2 of Desmoglein-4	5	3
B4DR52	Histone H2B	2	2
Q86TY5	*Galectin	3	2
P47929	Galectin-7	3	3
P13647	*Keratin, type II cytoskeletal 5	1	3

*Proteins identified in the present method but not found in the data published by Lee and co-workers [9].

## Discussion

Because hair deteriorates extremely slowly, it has been widely used in forensic investigations since the last century. Aside from that, other utilities of this easily obtained and chemically stable biological specimen were seldom described. The currently available methods for extraction of proteins from hair samples are time consuming and complex due to the poor solubility of hair proteins and the difficulty in their extraction caused by presence of highly cross-linked disulfide linkages [3]. In the present study, an alkaline lysis buffer, similar to that utilized for the purification of plasmids from chromosomal DNA, was formulated for extraction of proteins from the human hair shaft [14].

The effect of sodium dodecyl sulfate (SDS) on protein loss during extraction has been previously investigated [15]. The study showed that loss of protein was roughly two times higher in presence of SDS than in water, and more loss was observed when the temperature was increased. In the present study, NaOH was used to break the disulfide bonds of hair proteins but it was only workable in the presence of SDS. Our study also showed that proteins were not extracted in presence of SDS or NaOH alone. Hence, in this case, SDS may have facilitated the action of NaOH by popping holes in the hair cell membranes [16]. The extracted proteins were then dissolved in sample buffer to maintain stability and kept at 4°C to avoid reformation of disulfide bonds [17]. However, the use of strong alkali in the present study may also result in deamidation of Asn and Gln residues of proteins. Both Asn and Gln deamidate by direct hydrolysis and proceeds through a glutarimide intermediate at alkaline or neutral conditions [18]. This activity needs to be analysed separately in future work.

The present study also demonstrated that heating is essential for increasing the recovery of proteins. During the first extraction, heating of hair shaft fragments for a longer period of time was able to increase the yield of proteins from the hair, to probably include those which were more resistant to heat [19]. However, by 30 minutes, a plateau was reached and further heating for 40 minutes did not result in more yields. Despite result of statistical analysis showed no significant difference (p < 0.05) in the protein recovery from 20–30 minutes and 30–40 minutes of incubation, the optimal time of incubation in this study was selected at 30 minutes due to highest protein recovery obtained.

Our study had also shown that a second extraction, which involved the isolation of proteins from the leftover hair fractions, was necessary to maximize recovery of proteins from the human hair shaft. However, this was only possible when the concentration of NaOH from the same alkaline lysis buffer used in the first extraction was reduced from 0.2 to 0.1 M. Other than that, heating was also avoided in the second extraction to prevent occurrence of hydrolysis. To further maximize the efficiency of extraction of proteins in the second stage of extraction, a mechanical force by magnetic stirring at room temperature was found to be necessary.

Our newly developed protocol, when compared with the previously reported Shindai method [5] and the method developed by Lee et al. [9], has not only resulted in increased recovery of proteins from the hair shaft, but was also able to shorten the duration of extraction. When compared to the Shindai method [5], the present method was approximately three times more efficient, based on the total percent recovery of proteins. The extraction time required was reduced from at least 24 h to 1.5 h. Moreover, an additional of 24 h of incubation using the Shindai method [5] also did not result in higher percent recovery.

To completely dissolve the protein pellets for analysis by SDS-PAGE, a few sample buffers were tested. Our study showed that the buffer containing urea was the sole sample buffer that was able to dissolve the pellets completely. This is most likely attributed to the high stability of keratins that prevents their solubility as well as digestibility by proteolytic enzymes and protein solvents. A study by Speakman [20] has demonstrated the importance of the lateral links between parallel polypeptide chains, the bond of the disulfide group of cysteine, and the polar link of diamino and dicarboxylic acids, which contribute to the stability of the keratin molecule.

Our QToF LC/MS analysis was able to identify a total of 60 proteins extracted from 5 mg of hair shaft of an individual using the alkaline lysis protocol. More proteins were detected from the second stage when compared to the first stage of human hair shaft protein extraction. Some of the proteins (Table 4b) were exclusively found in the second stage of extraction. These proteins could only be extracted when a mechanical force was applied to the hair which was immersed in fresh alkaline lysis buffer. On the other hand, keratin such as keratin 83 (Accession no. A0JNT2) and keratin 37 (Accession no. Q6A165) were identified only from the first stage of protein extraction. The finding that some proteins were only present in either the first or second extractions of hair suggests that they have extensive interactions with other cellular components such as those in intercellular junctions and the nuclear periphery [21]. Proteins that are present in the cortex of hair could be easily extracted without solubilizing the cell borders [22]. Therefore, keratins that were found in the second stage of extraction were most likely to originate from the hair cuticle and medulla.

When compared to the study by Lee and coworkers [9] where over hundreds of proteins were identified, only 60 proteins were identified in our study. However, the majority of the proteins identified by Lee et al. using MudPIT was mainly from the non-solubilised (crossed-linked) fraction, whilst our study performed by QToF analysis had focused on the solubilised proteins. The use of MudPIT method in the study of Lee and coworkers has been proven to be a powerful high-throughput tool for separation of complex peptide mixtures [23]. MudPIT was also found to generate better results even for a relatively simple sample compared to a single dimension LC method [24]. Despite the variation in techniques used for protein identification, our present study which separated proteins based on their sizes by SDS-PAGE, was able to identify 25 proteins that were not previously reported in the data by Lee and coworkers. The detection of these proteins could be due to the different extraction method and/or improvements of the database. Among the 60 proteins identified by our QToF LC/MS analysis, 31 proteins identified were keratins, 28 were non-keratins while only one keratin-associated protein was found. In our study, we had detected the presence of galectin and galectin-7 in the human hair shaft. Although this appears to be an improvement over the method of Lee [9] which only detected galectin-7, it may also be because of hair sample variability. Aside from the human hair shaft, galectin-7 is known to be expressed in interfollicular epidermis of esophagus, oral epithelia, cornea and Hassall’s corpuscles of the thymus, and hence, can be considered as a marker of all subtypes of keratinocytes [25]. A previous study by Wollina and co-workers [26] had detected the expression of galectins-1 in the outer and inner root sheaths of murine hair. These endogenous lectins were found to be greatly hair cycle-dependent. However, the study of a cycle-related expression of galectin-1 and its binding sites during the human hair cycle has not been reported.

## Conclusions

In the present study, an alkaline lysis method of extraction of hair shaft proteins was newly developed. Substantially higher percentage of protein recovery was achieved using the method as compared to the previously reported Shindai method [5] and the protocol of Lee et al. [9]. Aside from being simple and reproducible, the time of extraction using the alkaline lysis method was reduced from five days or 48 hours to only 90 minutes. The present study has also identified 60 proteins using QToF analysis, with 25 proteins not reported in the previous data by Lee and coworkers [9]. This may open the door to more in depth studies on the basic protein analysis of the human hair shaft, disease markers and the study of molecular pathways that could possibly lead to novel therapeutic targets.

## Supporting Information

S1 Table Protein recovery measured by Bradford colorimetric assay at different time intervals. (DOC)

S2 Table Raw data of percent recovery obtained from both stages of protein extraction for all ten individuals. (DOCX)

## Abbreviations

SDS-PAGE: sodium dodecyl sulfate polyacrylamide gel electrophoresis
QToF: quadrupole-time-of-flight
KAPs: keratin associated proteins
NanoLC-MS/MS: nanoscale liquid chromatography coupled to tandem mass spectrometry
SDS: sodium dodecyl sulfate
DTT: dithiothreitol
MudPIT: multidimensional protein identification technology
βME: beta-mercaptoethanol
EDTA: ethylenediaminetetraacetic acid
CHAPS: 3-[3-Cholamidopropyl)dimethylammonio]-1-propanesulfonate
Tris-HCl: tris-hydrochloric acid
HPLC: high performance liquid chromatography
%SPI: scored peak intensity
FDR: false discovery rate
