# Supplementary material for: A High-Yield Two-Hour Protocol for Extraction of Human Hair Shaft Proteins
Source: PLoS One. 2016 Oct 14;11(10):e0164993. doi: 10.1371/journal.pone.0164993 (PMC5065217; doi:10.1371/journal.pone.0164993)
Supplement: S1 Table — (DOC) [file pone.0164993.s001.doc]

| **S1 Table. Protein recovery measured by Bradford colorimetric assay at different time intervals.** | | | | | | |
| --- | --- | --- | --- | --- | --- | --- |
| **Volunteer** | **Protein recovery (%)** | | | | | |
|  | **Time (min)** | | | | | |
|  | **0*** | **5** | **10** | **20** | **30** | **40** |
| **1** | 2.088 | 6.72 | 26.768 | 36.256 | 38.472 | 35.704 |
| **2** | 4.1832 | 25.8424 | 31.8968 | 26.8624 | 33.9616 | 30.7992 |
| **3** | 5.9072 | 6.676 | 11.5416 | 16.2376 | 33.1208 | 27.72 |
| **4** | 0.8936 | 10.084 | 13.3128 | 28.3312 | 34.96 | 29.4088 |
| **5** | 2.624 | 10.88 | 26.232 | 30.704 | 35.112 | 35.704 |
| **6** | 1.5104 | 3.4496 | 14.6144 | 31.6488 | 33.8264 | 35.4896 |
| **7** | 3.64 | 26.312 | 33.352 | 35.648 | 35.536 | 37.956 |
| **8** | 1.008 | 4.272 | 13.272 | 25.096 | 25.48 | 28.424 |
| **9** | 2.336 | 14.169 | 26.704 | 32.36 | 38.217 | 38.0128 |
| **10** | 3.1276 | 9.39 | 16.477 | 25.336 | 29.4002 | 27.2886 |
| **Average** | 2.7318 | 11.7795 | 21.3737 | 28.848 | 33.8086 | 32.6507 |

*minute 0 acts as the control of the experiment which the incubation mixture was left at room temperature for 60 minutes
