# Supplementary material for: A High-Yield Two-Hour Protocol for Extraction of Human Hair Shaft Proteins
Source: PLoS One. 2016 Oct 14;11(10):e0164993. doi: 10.1371/journal.pone.0164993 (PMC5065217; doi:10.1371/journal.pone.0164993)
Supplement: S2 Table — (DOCX) [file pone.0164993.s002.docx]

| **S2 Table. Raw data of the percent recovery obtained from both stages of protein extraction for all ten individuals.** | | | |
| --- | --- | --- | --- |
| **Individual** | **Percent recovery (%)** | | **Total percent recovery (%)** |
|  | **First stage of extraction** | **Second stage of extraction** |  |
| 1 | 28.12 | 10.316 | 38.436 |
| 2 | 29.04 | 8.430 | 37.47 |
| 3 | 25.029 | 9.54 | 34.569 |
| 4 | 26.21 | 5.9392 | 32.1492 |
| 5 | 52.513 | 8.68 | 61.193 |
| 6 | 33.704 | 26.290 | 59.994 |
| 7 | 35.603 | 11.53 | 47.133 |
| 8 | 31.4224 | 21.551 | 52.9734 |
| 9 | 30.22 | 14.28 | 44.5 |
| 10 | 46.38 | 17.84 | 64.22 |
| Average | 33.82414 | 13.43962 | 47.26376 |
